# Fault-Tolerant Network-On-Chip Router Architecture Design for Heterogeneous Computing Systems in the Context of Internet of Things

**DOI:** 10.3390/s20185355

**Published:** 2020-09-18

**Authors:** Muhammad Rashid, Naveed Khan Baloch, Muhammad Akmal Shafique, Fawad Hussain, Shahroon Saleem, Yousaf Bin Zikria, Heejung Yu

**Affiliations:** 1 Department of Computer Engineering, University of Engineering & Technology, Taxila 47050, Pakistan; rashid.khan9552@gmail.com (M.R.); naveed.khan@uettaxila.edu.pk (N.K.B.); fawad.hussain@uettaxila.edu.pk (F.H.); shahroon.saleem@uettaxila.edu.pk (S.S.); 2 Department of Electrical Engineering, University of Engineering & Technology, Taxila 47050, Pakistan; akmal.shafique@yahoo.com; 3 Department of Information and Communication Engineering, Yeungnam University, Gyeongsan-si 38541, Korea; 4 Department of Electronics and Information Engineering, Korea University, Sejong 30019, Korea

**Keywords:** IoT, heterogeneous computing systems, fault tolerance, network-on-chip, router architecture, permanent fault

## Abstract

Network-on-chip (NoC) architectures have become a popular communication platform for heterogeneous computing systems owing to their scalability and high performance. Aggressive technology scaling makes these architectures prone to both permanent and transient faults. This study focuses on the tolerance of a NoC router to permanent faults. A permanent fault in a NoC router severely impacts the performance of the entire network. Thus, it is necessary to incorporate component-level protection techniques in a router. In the proposed scheme, the input port utilizes a bypass path, virtual channel (VC) queuing, and VC closing strategies. Moreover, the routing computation stage utilizes spatial redundancy and double routing strategies, and the VC allocation stage utilizes spatial redundancy. The switch allocation stage utilizes run-time arbiter selection. The crossbar stage utilizes a triple bypass bus. The proposed router is highly fault-tolerant compared with the existing state-of-the-art fault-tolerant routers. The reliability of the proposed router is 7.98 times higher than that of the unprotected baseline router in terms of the mean-time-to-failure metric. The silicon protection factor metric is used to calculate the protection ability of the proposed router. Consequently, it is confirmed that the proposed router has a greater protection ability than the conventional fault-tolerant routers.

## 1. Introduction

Applications that improve the lifestyles of users such as the Internet of things (IoT), cloud computing, and cognitive computing have attracted considerable attention in recent years [1,2]. These applications and systems generate enormous amounts of data continuously [3]. They require exascale computing systems to process these continual data. Exascale computing systems have high capabilities of computation and storage, with several heterogeneous cores on a chip [4]. A previous study investigated the design of fault-tolerant capabilities for industrial cyber–physical systems (ICPS) and real-time monitoring using efficient hardware infrastructure [5]. The authors emphasized the use of artificial intelligence and deep learning and the development of new fault-tolerant techniques to contribute to the design and development of ICPS. These heterogeneous cores require scalable and high-performance communication infrastructure to exchange data. Consequently, network-on-chip (NoC) architectures have been developed [6,7]. NoC architectures provide scalability in terms of bandwidth and topology [8]. NoC is acknowledged as a “super highway” for the Intel Xeon scalable processor for Intel data centers [9]. The performance of a heterogeneous computing system is determined by not only the on-chip resources but also the on-chip communication. Therefore, NoCs are crucial for the reliability and performance of these systems.

Owing to aggressive scaling in process technology, transistors have become more prone to both transient and permanent faults [10]. Transient faults occur for a short period of a few cycles. They occur because of process variation [11], cosmic rays [12], and alpha particle strikes from the packaging material [13]. In contrast, permanent faults occur perpetually after their first appearance. They occur for two main reasons. The first is the increasing complexity of the fabrication process, which results in a higher rate of post-manufacturing faults. The second is the decreasing feature size of transistors, which causes hot carrier injection (HCI) [14], time-dependent dielectric breakdown (TDDB) [15], and negative-bias temperature instability (NBTI) [16]. Post-manufacturing faults are controlled by improving the fabrication process, whereas the effects of defects such as HCI, TDDB, and NBTI are not reversible.

A typical NoC comprises interconnected routers and attached processing elements (PEs) [17]. A single permanent fault in the router may cause performance degradation, disconnect a PE from the network, or even cause failure of the entire system. The router is the most important component of NoC, as it interconnects all the PEs and provides a communication infrastructure. Thus, it is imperative to design a permanent fault-tolerant router for a reliable network operation.

This paper presents a fault-tolerant NoC router architecture that protects all the components of the router against permanent faults. It tolerates multiple faults in the input port, route computation (RC), virtual channel allocation (VA), switch allocation (SA), and crossbar (XBAR) units. In detail, the input port utilizes a bypass path and virtual channel (VC) closing strategies to tolerate faults in multiplexers, de-multiplexers, and VCs. The RC stage uses spatial redundancy and double routing strategies to tolerate an RC unit failure. The first stage of VA utilizes spatial redundancy for arbiter failure. The second stage of VA employs a combined allocation of VA and SA to mask arbiter failure. The SA stage uses run-time arbiter selection. Additionally, the XBAR stage utilizes a triple bypass bus strategy to bypass a faulty XBAR. Owing to these features, the proposed router has a low area overhead and tolerates a high number of faults compared with the state-of-the-art fault-tolerant router.

The remainder of this paper is organized as follows. Section 2 discusses the state-of-the-art fault-tolerant NoC router architectures. Section 3 describes the proposed fault-tolerant NoC router architecture. Section 4 and Section 5 present the latency and reliability analyses, respectively. Finally, Section 6 concludes this paper.

The main contributions of this study are as follows:

- This study utilizes the inherent redundancies in the pipeline and lookahead routing to maintain the performance in the presence of faults.
- This study proposes highly fault-tolerant schemes for each stage of the router pipeline.
- This study compares the latency, hardware consumption, and reliability of the proposed architecture with those of the state-of-the-art fault-tolerant router architectures.

## 2. Related Work

In [18], the authors emphasized the fault diagnosis and fault-tolerant control problem of Markov jump systems (MJS), which exist abundantly in mobile manipulator systems. They investigated the fault-tolerant control for MJS sensor faults. The developed system model is based on stochastic noise terms and the time delay of the state variables and results in the characterization of more features than the current designs. Industry 4.0 and ICPS are the core concepts of IoT. In [19], the authors investigated and reviewed the monitoring, fault diagnosis, and control tasks related to ICPS. Moreover, unobservable attacks, problems in data-driven assessment, and fault-tolerant schemes were discussed in detail. A cyber–physical system comprises embedded sensors and actuators for an interaction with the environment. The availability of the Internet has improved the scalability and functionalities of such systems, but they are susceptible to security threats. Therefore, it is necessary to avoid such threats using cyber security techniques. In [20], the authors analyzed the impact of replay attacks on ICPS. They proposed that the tolerance against replay attacks can be improved by adding an authentication signal to the control unit. Cyber security for power systems has been studied extensively; researchers have focused on several cyber–physical attacks, but they have rarely considered availability attacks. In [21], the authors modeled a hybrid cyber attack by combining availability and integrity attacks. They examined false-negative and false-alarm attack scenarios and proved that the proposed model lowers the attacks with a reduced cost.

In [22], the authors proposed the BulletProof router. It utilizes N-modular redundancy (NMR) and error correcting codes (ECCs) to protect the components of the router against permanent faults. However, NMR has a large area overhead. The RoCo router investigated in [23] is divided into horizontal and vertical modules. It uses parallel arbiters and smaller XBARs for horizontal and vertical connections. The horizontal and vertical modules perform independently. Thus, a defective horizontal module does not disturb the operation of the vertical module, and vice versa.

In [24], the authors proposed the Vicis router. It tolerates permanent faults at the network and router levels. The network-level faults use input port swapping and adaptive routing algorithms. On the other hand, the router-level faults use ECC and bypass the bus to tolerate permanent faults in the input buffers and XBAR, respectively. In [25], the REPAIR router was investigated. It improves the input port swapping algorithm of the Vicis router through expensive re-routing. However, it incurs an area overhead of 50%, which is higher than that of the Vicis router (40%).

In [26], the authors proposed a PVS router. It exploits a partial VC-sharing strategy to tolerate the faults in the input port and RC unit. However, if the shared component receives a fault, all the associated input ports become inaccessible. Accordingly, the decoupled resource sharing (DRS) router, which shares the resources of three adjacent input ports through DRS modules, was proposed to overcome this problem [27]. Even when the DRS module of an input port becomes faulty, it does not disturb the functionality of the neighboring input ports.

In [28], the authors proposed the SHIELD router. It tolerates permanent faults in all the pipeline stages. The RC unit employs spatial redundancy. The VA unit employs resource sharing. The SA unit uses the default winner strategy. The XBAR unit employs multiple secondary bypass paths. Additionally, the NoCGuard router was proposed in [29]. It also tolerates permanent faults in all the pipeline stages. The RC unit employs resource sharing and double-routing strategies. The VA unit uses the default winner strategy. The SA unit employs run-time arbiter selection and default winner strategies. The XBAR unit uses multiple secondary bypass paths. Moreover, this router tolerates a higher number of faults in each pipeline stage than the SHIELD router at a reduced cost.

In [30], the authors proposed high performance router (HPR). It tolerates faults in all the components of the router. The input port buffers employ ECC. The RC unit employs a dual routing scheme. The VA unit employs the default winner strategy. The SA unit employs run-time arbiter selection strategy. The XBAR unit employs a double bypass bus strategy. In [31], the authors proposed the Defender router. It also tolerates faults in all the components of the router. The input port employs resource sharing. The RC unit employs the default winner strategy. The VA unit employs the default winner strategy. The SA unit employs run-time arbiter selection strategy. The XBAR unit provides two bypass buses that bypass a faulty crossbar. Moreover, this router tolerates more faults than the HPR router.

In [32], the authors proposed NoCAlert. It is a comprehensive online fault detection mechanism for NoC router architectures. It comprises multiple checkers for each router component. They seamlessly and concurrently monitor functional irregularities. NoCAlert detects 97% of router faults. This fault detection mechanism incurs area and power overheads of 0.3% and 0.7%, respectively.

The proposed fault-tolerant router detects and tolerates permanent faults in all the components of the router. It uses NoCAlert for fault detection [32]. The proposed router tolerates a higher number of faults in all the components of the router than existing state-of-the-art fault-tolerant routers.

## 3. Proposed Fault-Tolerant NoC Router Architecture

**Figure 1:**
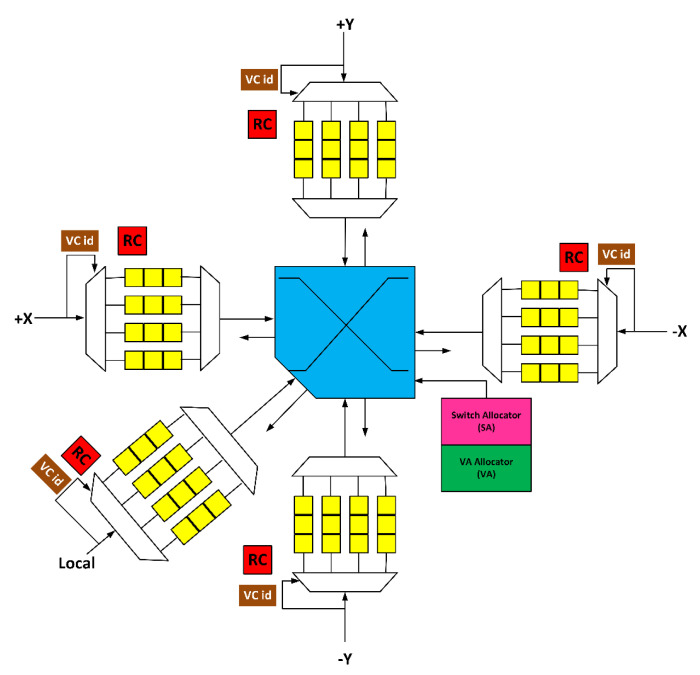
Baseline network-on-chip (NoC) router micro architecture.

Figure 1 depicts the baseline NoC router. It comprises five input ports, five output ports, and four pipeline stages, namely, RC, VA, SA, and XBAR [33].

**Figure 2:**
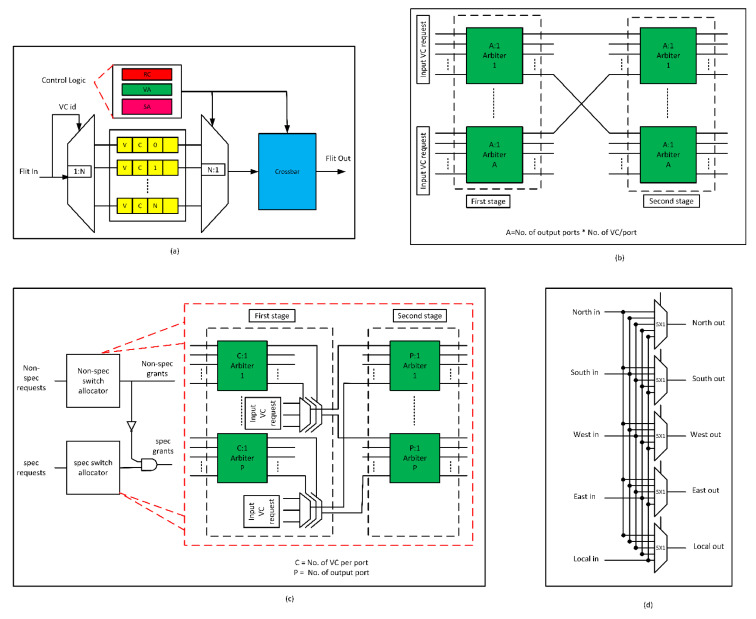
Pipeline stages in router architecture: (**a**) input port, (**b**) virtual channel (VC) allocator, (**c**) switch allocator, (**d**) crossbar.

Each input port comprises de-multiplexers, multiplexers, and VCs, as shown in Figure 2a. The de-multiplexers and multiplexers guide the flits in and out of the VCs. Each input port has four VCs. Each VC comprises buffers and stores incoming flits.

The first pipeline stage comprises RC units that compute the route for the packet on its arrival. This stage operates only on the head flit. The baseline router employs lookahead routing, which computes the routing for the downstream router and embeds the result in the packet.

The second pipeline stage comprises the VA unit, which allocates an empty VC buffer for each packet at the downstream router. VA comprises two stages of arbiters, as shown in Figure 2b. In the first stage of VA, the input VC with a head flit competes with other VCs for an empty VC in the downstream router. The second stage arbitrates among the input VCs winning arbitration for the same downstream VC.

The third pipeline stage comprises the SA unit, which grants the flits access to the XBAR. It is a two-stage process, as shown in Figure 2c. The first stage arbitrates among the input port VCs attempting to access the XBAR. The second stage arbitrates among the input ports winning arbitration for the same input port of the XBAR. The baseline router comprises two separable SA units: non-speculative and speculative SA. Speculative SA occurs in parallel with VA. The flits that win both VA and speculative SA simultaneously traverse the XBAR in the next cycle. The flits that do not win VA or speculative SA proceed to arbitration through non-speculative SA in the next cycle.

The fourth pipeline stage comprises the XBAR unit, which allows the flits in the input to access the output ports. The baseline router comprises a multiplexer-based 5x5 XBAR, as shown in Figure 2d. The SA stage provides control signals to reconfigure multiplexers every cycle.

Each component of the router pipeline performs a distinct role in the operation of the router. The functionality of each pipeline component depends on the results of the previous component. Thus, it is necessary to protect all the components against permanent faults.

### 3.1. Fault-Tolerant Design of Input Port

**Figure 3:**
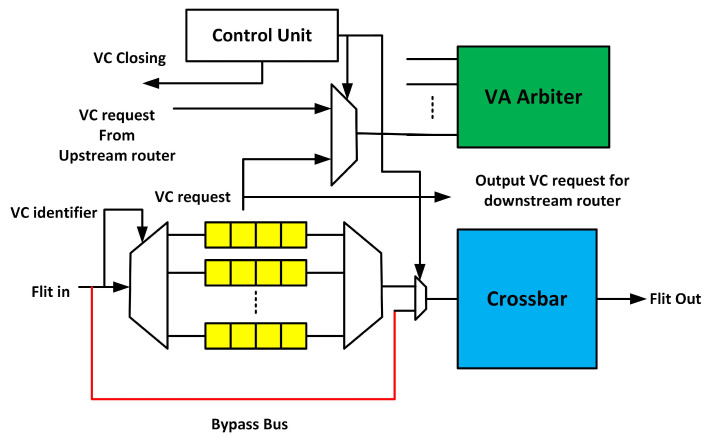
Input port fault-tolerant design.

The input port comprises a de-multiplexer, multiplexer, and VCs. The permanent faults in the de-multiplexer and multiplexer block flits arriving in and out of VCs. If a permanent fault occurs inside the VC, it can corrupt the flits. Thus, it is necessary to bypass or mask the effects of these faults. We propose a bypass path with the VC queuing and closing mechanism, which maintains the functionality of the router even if all the de-multiplexers, multiplexers, and VCs become faulty. Figure 3 shows the modified input port. When a fault occurs in the VC, the control unit sends the signal to the upstream router to stop sending flits to the faulty VC. Hence, this VC closes. Upon the failure of the four VCs, multiplexer, or de-multiplexer, the entire port becomes faulty. Then, the bypass path is activated. Flits use the bypass path to reach the XBAR. The flow of these flits is still controlled by the current router. The upstream router stops the flit until it wins the VA and SA in the current router. Thus, flits are physically stored in the upstream router, but are virtually queued and arbitrated in the current router through control signals between adjacent routers. As they win both the VA and SA stages, they traverse the bypass path to reach the XBAR and, finally, their destination. This technique maintains the functionality of the router even if the de-multiplexer, multiplexer, and all the VCs of the input port become faulty.

### 3.2. Fault-Tolerant Design of RC Stage

**Figure 4:**
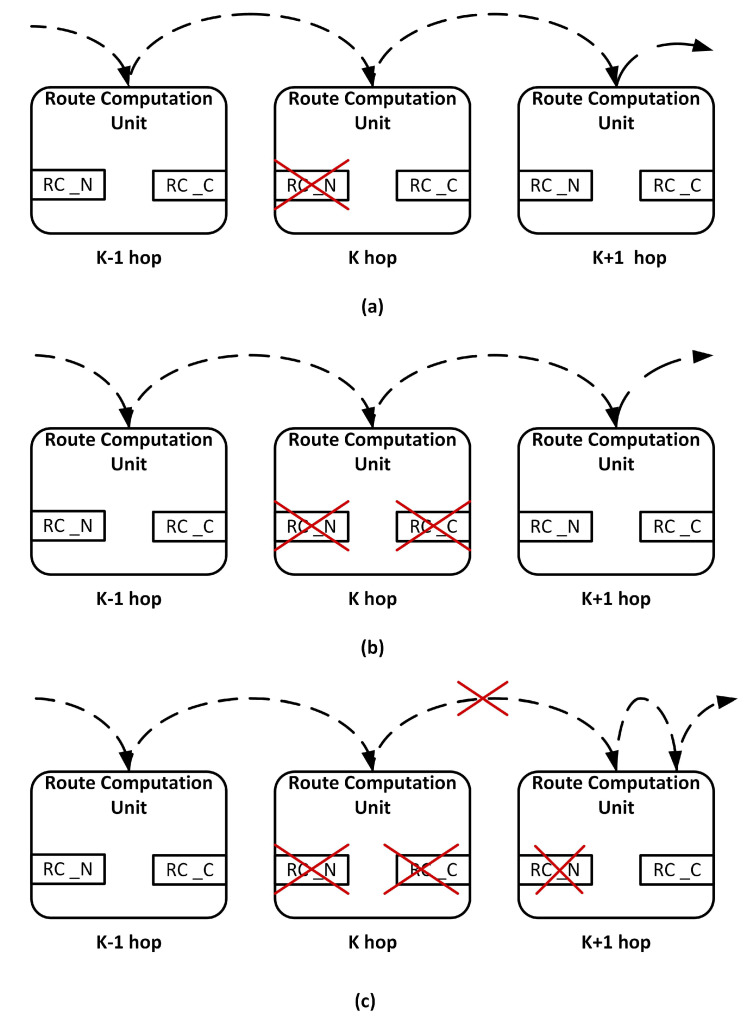
Route computation (RC) fault-tolerant design: (**a**) scenario 1 where RC_N is faulty, (**b**) scenario 2 where both RCs are faulty, and (**c**) scenario 3 where both RCs of the current and RC_N of downstream router are faulty.

Each input port has its own RC unit. A permanent fault causes deadlock or misroute flits. The baseline router employs the lookahead routing mechanism. Accordingly, the faulty RC unit computation does not cause misrouting in the current router. Misrouting occurs in the downstream router, which utilizes this computation. We utilize a double routing strategy and provide a redundant RC per input port to handle this situation. Figure 4 shows the modified RC stage. RC_N represents the RC unit that computes the lookahead route in a fault-free scenario. RC_C represents the redundant RC unit. In the case of a fault, the following scenario arises:

#### 3.2.1. Scenario 1

If the RC_N unit becomes faulty, the RC_C unit replaces it. Now, the RC_C unit computes the lookahead route. Figure 4a depicts this scenario.

#### 3.2.2. Scenario 2

If both the RC_N and RC_C units become faulty, the packet in the downstream router is blocked. To handle this situation both the RC units in the downstream router are activated. The RC_N unit computes the lookahead route, whereas the RC_C unit computes the current route. Figure 4b depicts this scenario.

#### 3.2.3. Scenario 3

If both the RC_N and RC_C units of the current router and the RC_N unit of the downstream router have a fault, then the RC_C unit of the downstream router performs the operation using a double routing strategy. It first computes the current route for a packet. When the packet is in the VA and SA stage, it computes the lookahead route. Figure 4c depicts this scenario.

**Figure 5:**
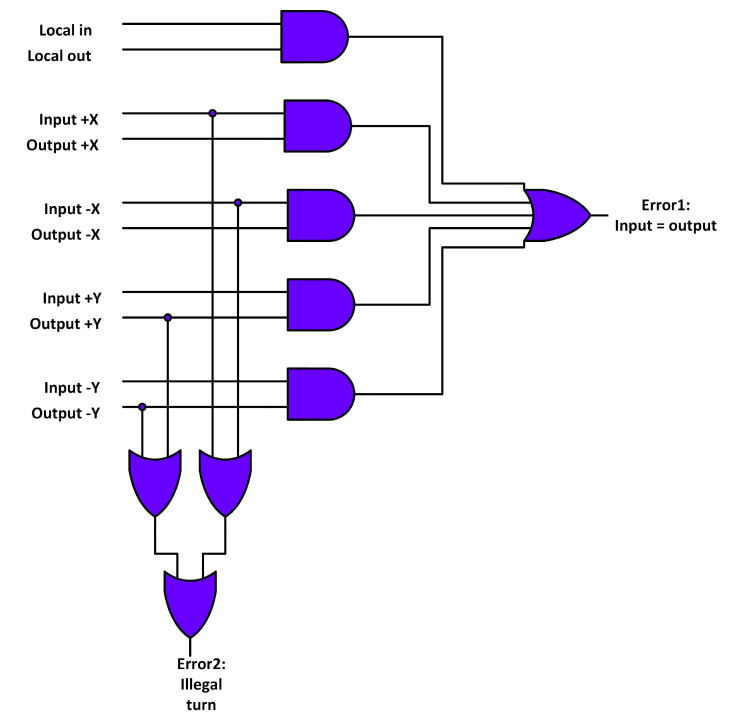
RC fault detection mechanism.

The NoCAlert checkers [32] were used to detect faults in the RC unit. Figure 5 shows the RC fault detection mechanism. Error 1 signal asserts when the input and output ports of the flit are the same. Error 2 signal asserts when flit from north or south input ports turns to east or west output ports. Both cases violate the working principle of the XY routing algorithm.

### 3.3. Fault-Tolerant Design of VA Stage

VA occurs in two stages: VA1 and VA2. In VA1, there is an A:1 arbiter for every input VC. In VA2, there is an A:1 arbiter for every output VC, where A represents (the number of VCs per output port) × (the number of output ports), that is, 20. We separately consider fault tolerance in both stages.

#### 3.3.1. First Stage of VA (VA1)

**Figure 6:**
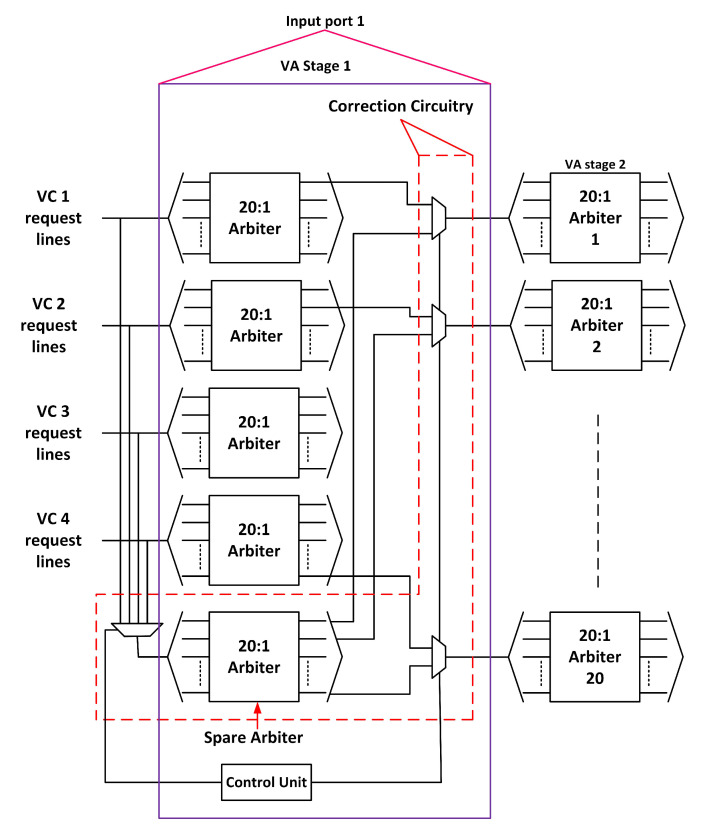
Virtual channel allocation stage 1 (VA1) fault-tolerant design.

To handle a faulty arbiter in VA1, we propose adding a spare arbiter for every input port. Figure 6 shows the modified VA1 stage. The request lines from all four VCs of an input port connect to the spare unit through a 4:1 multiplexer. The output of the spare arbiter connects to the corresponding arbiters in VA2 through a 2:1 multiplexer. The control unit generates the necessary signals in case of a fault. When an arbiter becomes faulty, the control unit routes the request lines to the spare arbiter. It allocates an output VC to the input VC. When two or more input VC arbiters become faulty, the spare unit operates in a round-robin manner. The control unit assigns the request lines to the spare unit in a round-robin manner. This technique functions well even if all the arbiters of an input port become faulty.

#### 3.3.2. Second Stage of VA (VA2)

**Figure 7:**
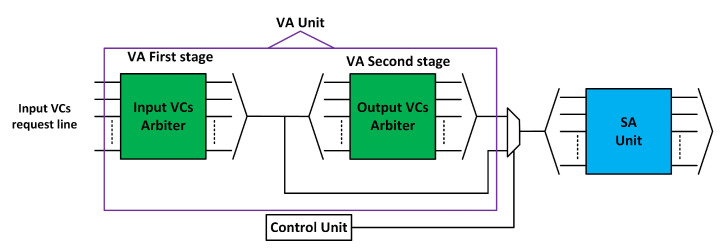
VA2 fault-tolerant design.

We employ pipeline optimization to mask the effect of a faulty arbiter in VA2. In [25], the authors proposed combining allocation that removes VA2 from the pipeline of the flit. Figure 7 shows the proposed scheme for VA2. From SA1, we observe that only one VC can send a flit at a time (the input VCs of the same input share the input of the XBAR). SA2 selects only one flit for an output port from multiple requests. This is a structural restriction imposed by the data path of the router. Owing to these restrictions, a flit can be allocated to an output VC by performing VA1 in series with SA. The output VC can be successfully assigned to the input VC even when its associated arbiter becomes faulty.

**Figure 8:**
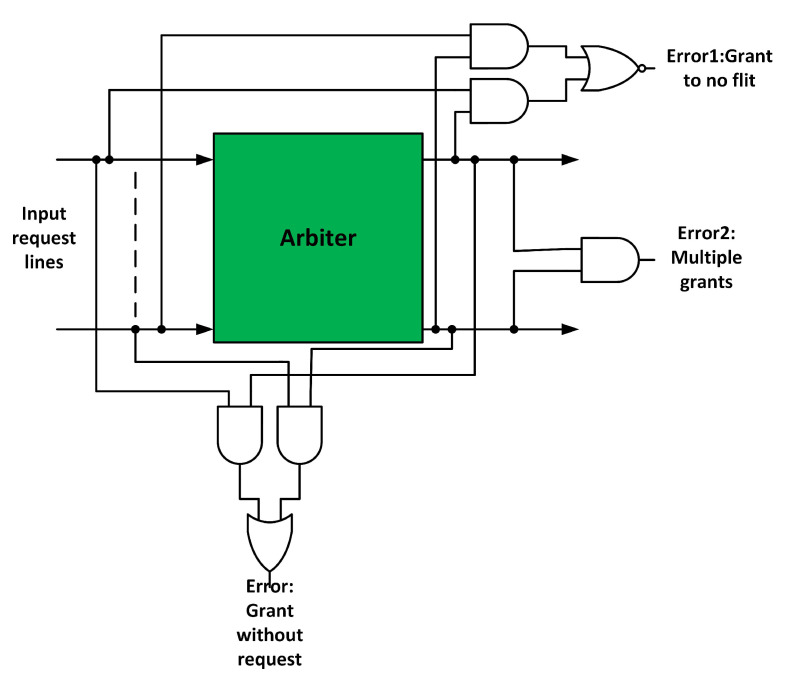
Arbiter fault detection mechanism.

The NoCAlert checkers [32] were used to detect faults in the arbiters. Figure 8 shows the arbiter fault detection mechanism. Error 1 signal asserts when one or more request lines are high, but the grant lines are zero. Error 2 signal asserts when multiple grants are detected. Error 3 signal asserts if the arbiter grants without a request. All these cases violate the working principle of the arbiter.

### 3.4. Fault-Tolerant Design of SA Stage

**Figure 9:**
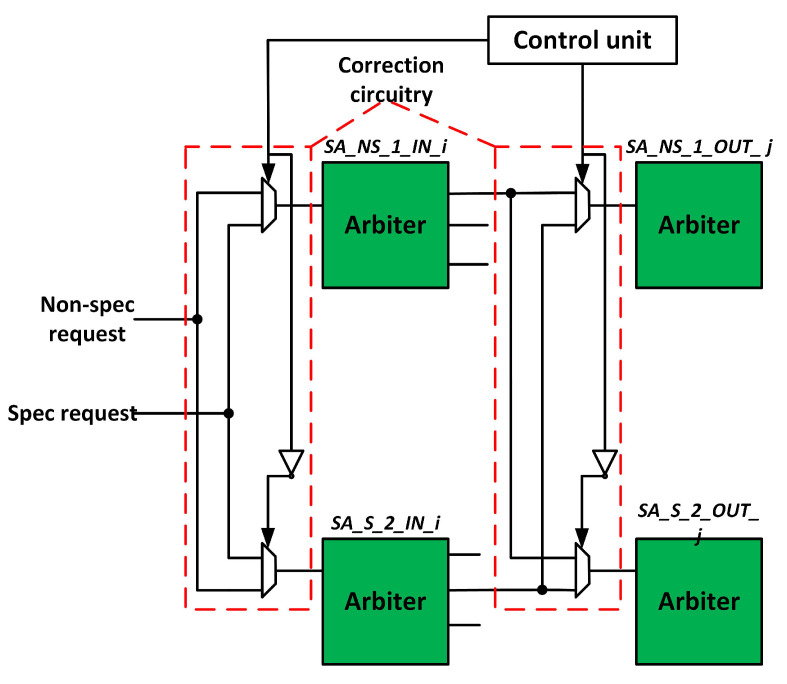
Switch allocation (SA) fault-tolerant design.

The baseline router comprises two similar sets of SA units: non-speculative and speculative SA. We exploit this redundancy to tolerate faulty arbiters. Figure 9 shows the modified SA stage. SA_NS_1 and SA_NS_2 handle non-speculative requests, whereas SA_S_1 and SA_S_2 handle speculative requests. If the arbiter in the non-speculative SA becomes faulty, its requests shift to a speculative SA arbiter. Now, speculative SA handles both types of requests. Both stages of SA units exploit this strategy. The proposed technique tolerates faults at runtime and avoids stalls.

### 3.5. Fault-tolerant Design of XBAR Stage

**Figure 10:**
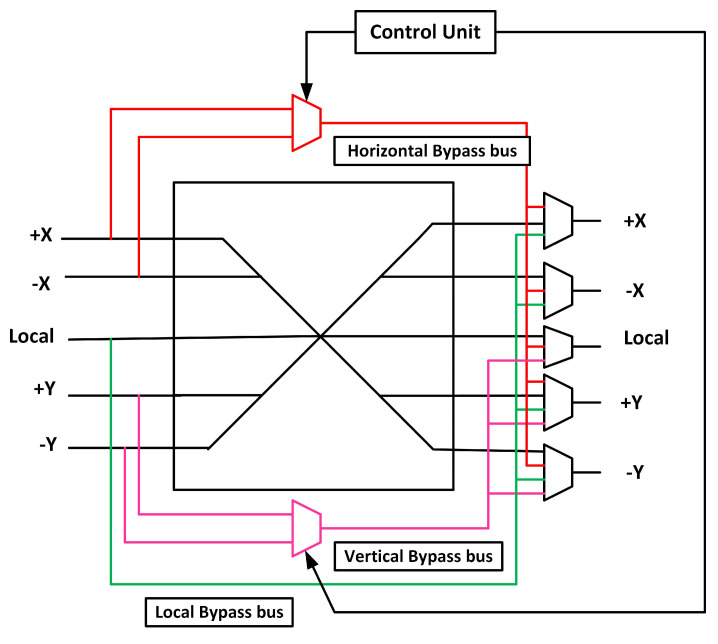
Crossbar (XBAR) fault-tolerant design.

XBAR connects the input and output ports of a router. If a fault occurs in an XBAR, flits cannot reach the output ports. We propose an XBAR with a triple bypass bus to make it fault-tolerant. Figure 10 shows the proposed fault-tolerant scheme for the XBAR.

We add three bypass buses to traverse across the faulty XBAR: horizontal, vertical, and local bypass buses. A horizontal bypass bus connects the X-dimension input ports to all the output ports. The vertical bypass bus connects the Y-dimension input ports to the Y-dimension and local output ports. In the XY routing algorithm, flits first traverse the X-dimension and then the Y-dimension. Thus, Y-dimension input ports do not connect to X-dimension output ports. The local bypass bus connects the local input port to all the output ports. The proposed XBAR traverses three flits at a time in a worst-case scenario, that is, if all the multiplexers of the XBAR are faulty.

## 4. Latency Analysis

We examine and compare the proposed router with the baseline router to analyze the effect of the fault-tolerant circuitry on latency. The Gem5 simulator [34] was used for the simulation. The proposed router was implemented in Garnet [35], which is a cycle-accurate NoC simulator integrated into Gem5. The simulation is performed on an 8×8 mesh network with four VCs per port. Each VC has 16 buffers of 128 bits. Synthetic and application benchmark traffic patterns are used for the simulation. The most effective method to simulate faults is to inject faults based on the failure in time (FIT) values of the component. The FIT values are minute and require applications to run for a long period of time. To speed up the simulation, we inject multiple permanent faults in the router components after 1 million cycles of its operation.

**Figure 11:**
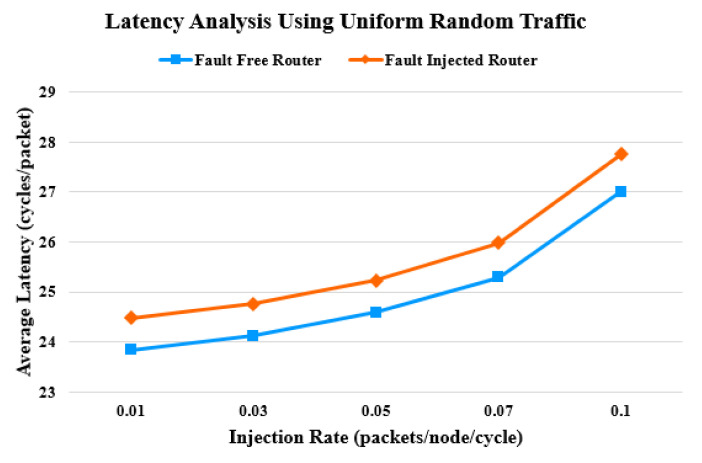
Latency comparison under uniform random traffic pattern.

**Figure 12:**
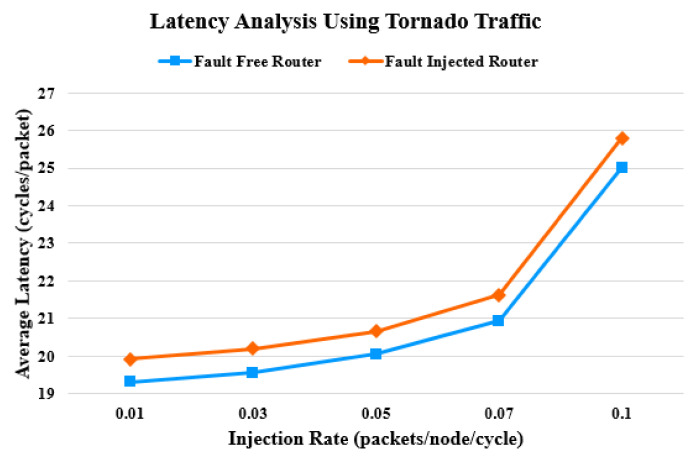
Latency comparison under tornado traffic pattern.

In the first part of the experiment, the synthetic traffic patterns are used for simulation. The simulation runs for five different injection rates. Figure 11 and Figure 12 show the latency assessment of the proposed router compared with that of the baseline unprotected router. As the injection rate approaches 0.1, contention increases. This causes latency to increase, as packets have to wait longer for resource allocation. Faults in the router pipeline aggravate the situation of delayed resource allocation and further contribute to an increase in latency. Beyond an injection rate of 0.1, latency increases exponentially. In a fault-free scenario, the proposed router consumes no additional cycles. When faults are injected into the proposed router, the average latency increases by 2.69 % and 3.17% for the uniform random and tornado traffic patterns, respectively.

**Figure 13:**
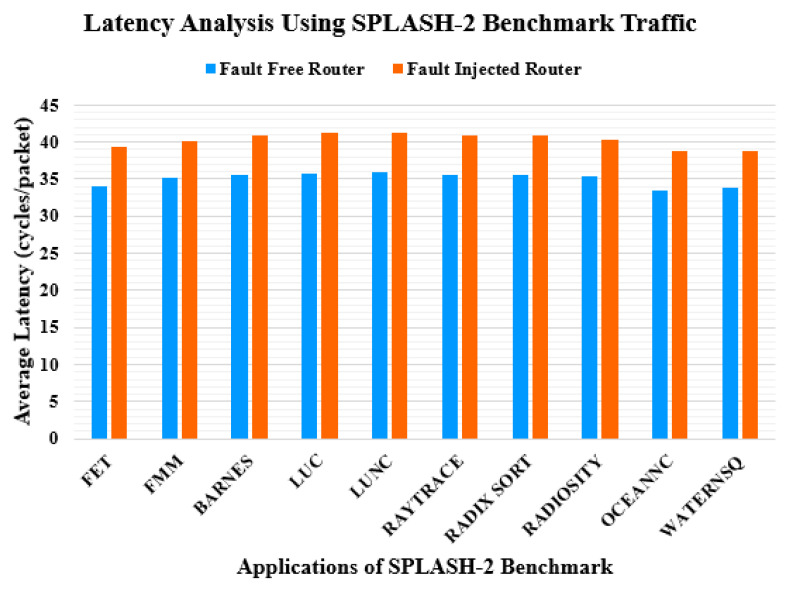
Latency comparison under SPLASH-2 benchmark traffic pattern.

**Figure 14:**
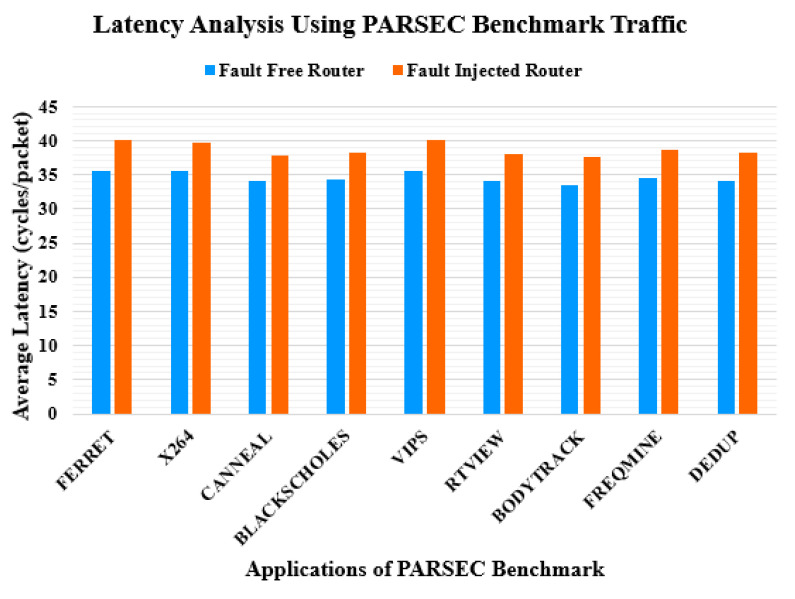
Latency comparison under PARSEC benchmark traffic pattern.

In the second part of the experiment, the application benchmark traffic patterns, that is, stanford parallel applications for shared-memory (SPLASH-2) [36] and princeton application repository for shared-memory computers (PARSEC) [37], are used for simulation. The configuration of the routers remains the same as in the first part of the experiment. Each core has its cache and directory. Figure 13 and Figure 14 show the latency assessment of the proposed router compared with that of the baseline unprotected router. The average latency of the proposed router increases by 15% and 12% for the SPLASH-2 [36] and PARSEC [37] benchmark traffic patterns, respectively.

## 5. Reliability Analysis

### 5.1. Hardware Overhead Analysis

The baseline router was first implemented in Verilog HDL for hardware overhead analysis. Then, NoCAlert fault detection checkers [32] were added to each pipeline component. Finally, the reconfigurable fault tolerance scheme for each pipeline component was implemented on top of the router. When there is no fault in the router, it behaves like the baseline router. In the presence of a fault in a pipeline component, the corresponding fault-tolerance circuitry becomes activated to perform router operation. For synthesis, we used the NangateOpenCell 15 nm technology library [38] with the Cadence Encounter RTL compiler. The synthesis results reveal that the proposed router consumes 26.6% more area and 28% more power than the baseline router.

### 5.2. Lifetime Reliability Analysis Using MTTF

We use the mean-time-to-failure (MTTF) [39] metric to evaluate the lifetime reliability of the proposed router compared with that of the baseline router. The MTTF of a component can be calculated as

(1) $$MTTF_{Component}=\frac{1}{FIT_{Component}},$$

where $FIT_{Component}$ is defined as the number of failures per billion hours of operation. To estimate $FIT_{Component}$, we use the FIT estimation model proposed in [40]. For the TDDB failure mechanism, the MTTF is given as

(2) $$MTTF_{Component}=N_{logic\_gate}\times D\times \frac{10^{9}}{A_{TDDB}}\times {\left(V_{dd}\right)}^{a-bT}\times e^{\frac{X+\frac{Y}{T}+ZT}{KT}},$$

where $A_{TDDB}$, *a*, *b*, *X*, *Y*, and *Z* are fitting parameters, whose values are derived in [41]. $N_{logic\_gate}$ is the transistor count of a logic gate, *D* is the duty cycle (100%), $V_{dd}$ is the operating voltage (1 V), *T* is the operating temperature (300 K), and K is the Boltzmann constant. The sum-of-failure-rate (SOFR) [42] model is utilized to calculate the FIT of a logic circuit. It assumes that the FIT of a logic circuit is the sum of the FITs of the individual gates.

#### 5.2.1. FIT Calculation for Baseline Router

The baseline router comprises five input ports. Each port comprises four VCs. Each VC store 16 flits. Each flit is 128 bits wide. A basic component of the input port is a D flip-flop. The RC unit comprises two comparators, one for each dimension. VA and SA comprise arbiters. The XBAR unit comprises multiplexers. Table 1 lists the fundamental component (FC), the FIT of each FC, the number of FCs, and the total FIT of each stage of the baseline router.

Table 2 lists the fundamental component (FC), the FIT of each FC, the number of FCs, and the total FIT of each stage of the correction circuitry.

#### 5.2.2. FIT Calculation for Correction Circuitry

- **Input Port:** It employs a 128-bit 2:1 multiplexer at each input port.
- **RC Stage:** It employs an additional RC unit at each input port.
- **VA Stage:** VA1 employs an additional 20:1 arbiter and 20-bit 4:1 multiplexer per input port. VA2 employs 40 2:1 multiplexers, 20 of which are used to tolerate the fault of VA1.
- **SA Stage:** It employs 20 2:1 multiplexers.
- **XBAR Stage:** It employs 2 128-bit 4:1 multiplexers, 3 128-bit 3:1 multiplexers, and 2 128-bit 2:1 multiplexers.

#### 5.2.3. MTTF Calculation

The MTTF of the baseline router is calculated by utilizing the SOFR model [42] as

(3) $$MTTF_{baseline\_router}=\frac{10^{9}}{20480+117+1468+215+4096}\approx 37913.25Hours$$

The proposed fault-tolerant router operates well as long as the underlying baseline router or correction circuitry is fault-free. The MTTF of a system having two components, i.e., baseline router and correction circuitry, with the failure rates $FIT_{1}$ and $FIT_{2}$, respectively, is expressed by utilizing the SOFR model [42] as

(4) $$MTTF_{proposed\_router}=\frac{10^{9}}{FIT_{1}}+\frac{10^{9}}{FIT_{2}}+\frac{10^{9}}{FIT_{1}+FIT_{2}},$$

where $FIT_{1}$ is the FIT of the baseline router calculated as 20480 + 117 + 1468 + 215 + 4096 = 26376, and $FIT_{2}$ is the FIT of the correction circuit calculated as 1024 + 117 + 271.5 + 32 + 2867.2 = 4311.7. By substituting these values in Equation (4), the MTTF is determined to be 302426.68 hours. It is 7.98 times higher than that of the baseline router. Thus, the lifetime reliability of the proposed router is 7.98 times higher than that of the baseline router.

### 5.3. Reliability Analysis using SPF

We use the silicon protection factor (SPF) [22] metric to compare the reliability of the proposed router with that of state-of-the-art fault-tolerant router architectures. SPF represents the amount of protection offered by the fault-tolerant system. The higher the SPF, the more resilient each transistor is to defects. The number of defects in a system is directly proportional to its area. Thus, SPF provides a representative notation of the fault tolerance provided by the proposed system. It is expressed as

(5) $$SPF=\frac{Average\;No.of\;Defects\;to\;Cause\;Failure}{\frac{Area\;of\;Fault-tolerant\;Router\;Architecture}{Area\;of\;Baseline\;Router\;Architecture}}$$

#### 5.3.1. Calculation of Average Number of Defects to Cause Failure

We first calculate the number of defects each component of the router tolerates to calculate the average number of defects that cause the failure of a router.

- **Input port:** The baseline router consists of five input ports. Each port consists of a de-multiplexer, multiplexer, and four VCs. The proposed fault-tolerant methodology tolerates faults in all the six components of an input port. Thus, a router tolerates a maximum of 30 input port defects. A defect in a de-multiplexer/multiplexer and bypass path causes the failure of an input port. Thus, a minimum of 2 defects cause input port failure.
- **RC Stage:** In the baseline router, each input port has its own RC unit. The proposed fault-tolerant methodology tolerates a maximum of 5 defects per router. If both the RC_N and RC_C units of adjacent routers become faulty, the RC fails. Thus, a minimum of 4 defects cause RC failure.
- **VA Stage:** The faults for the VA unit can be described as the fault in the arbiters. The number of arbiters in the first stage of VA is 20. The spare arbiter provides fault tolerance if all the arbiters in the first stage of VA are faulty. The second stage of VA also has 20 arbiters. Our pipeline optimization technique for fault tolerance also functions well if all the arbiters in the second stage are faulty. Therefore, our proposed technique can tolerate a maximum of 40 defects in a VA. A minimum of 2 defects cause failure if the original arbiter and the additional arbiter become faulty.
- **SA Stage:** The baseline router consists of two identical sets of SAs. If a defect occurs in a non-speculative arbiter, the corresponding arbiter in the speculative set performs SA. Thus, it tolerates a maximum of 10 defects. If both the corresponding arbiters fail, the SA fails. Thus, a minimum of 2 defects cause SA failure.
- **XBAR Stage:** The baseline router consists of a 5x5 crossbar. It has five multiplexers. The proposed triple bypass provides full functionality if all the multiplexers become faulty. Thus, it tolerates a maximum of 5 defects. If a bypass path and multiplexer become faulty, the XBAR fails. Thus, a minimum of 2 defects cause XBAR failure.

If a stage of the router fails, the entire router fails. We consider the smallest number among the minimum numbers of defects that cause the failure of each stage. Thus, minimum {2(Input port), 4(RC), 2(VA), 2(SA), 2(XBAR)} = 2, defects cause router failure. We add the maximum number of faults each stage tolerates to calculate the maximum number of faults the router tolerates. Thus, the router tolerates a maximum of total {30(Input port) + 5(RC) + 40(VA) + 10(SA) + 5(XBAR)} = 90, defects. Router will fail if one more defect occurs. Thus, maximum defects to cause router failure are 90 + 1 = 91.

The average number of defects that cause router failure is expressed as

(6) $$Average\;No.of\;Defects\;to\;Cause\;Failure=\frac{\begin{array}{c} Maximum\;No.\;of\;Defects\;to\;Cause\;Failure\;+\\ Minimum\;No.\;of\;Defects\;to\;Cause\;Failure \end{array}}{2}$$

Substituting the appropriate values yields

(7) $$Average\;No.of\;Defects\;to\;Cause\;Failure=\frac{91+2}{2}=46.5$$

#### 5.3.2. SPF Calculation

Considering the area overhead, the SPF value can be evaluated using Equation (5) as

(8) $$SPF=\frac{46.5}{1.26}=36.9$$

Table 3 presents the comparison of the SPF value of the proposed router with those of the state-of-the-art fault-tolerant router architectures. The proposed fault-tolerant architecture tolerates more faults incurring a minimum area overhead, compared with all the state-of-the-art fault-tolerant router architectures, by exploiting inter component dependencies and inherent redundancies. In addition, it achieves the highest SPF value compared with the state-of-the-art fault-tolerant router architectures. This indicates that the proposed router is more reliable than the state-of-the-art fault-tolerant router architectures.

## 6. Conclusions

NoC architectures are increasingly adopted in exascale heterogeneous computing systems owing to their scalability and performance. These systems are used in IoT applications, cognitive computing, and cloud computing. The reliability of NoC is one of the key issues. This paper proposed efficient techniques to improve the reliability of an NoC router against permanent faults. The proposed techniques provided fault tolerance for the input port, RC, VA, SA, and XBAR at the cost of modest additional circuitry. The hardware synthesis results revealed that the proposed router consumes 26.6% more area and 28% more power than the baseline router. The MTTF analysis showed that the reliability of the proposed router is 7.98 times higher than that of the baseline router. We used the SPF metric to estimate the protection ability of the proposed router. The result showed that the proposed router has a larger SPF value than that of the existing fault-tolerant router architectures and tolerates a greater number of faults in each router pipeline component. The idea of using the inherent redundancies in pipeline and adaptive algorithms can be used to design more reliable router architectures in the future.

## Figures and Tables

**Table 1:** Failure in time (FIT) values of the baseline router.

Stage	FC	FIT_FC_	# of FCs	FIT_Stage_
Input buffer	128-bit DFF	0.5	5x64	20480
RC stage	6-bit comparator	11.7	10	117
VA stage	20:1 arbiter	36.7	40	1468
	4:1 arbiter	7.4	10	215
SA stage	5:1 arbiter	9.3	10	
	4:1 multiplexer	4.8	10	
XBAR stage	128-bit 5:1 multiplexer	819.2	5	4096

**Table 2:** FIT values of the correction circuitry.

Stage	FC	FIT_FC_	# of FCs	FIT_Stage_
**Input buffer**	128-bit 2:1 mux	204.8	5	1024
**RC**	6-bit comparator	11.7	10	117
	20:1 arbiter	36.7	5	271.5
**VA**	4:1 multiplexer	4.8	5	
	2:1 multiplexer	1.6	40	
**SA**	2:1 multiplexer	1.6	20	32
	128-bit 4:1 multiplexer	614.4	2	2867.2
**XBAR**	128-bit 3:1 multiplexer	409.6	3	
	128-bit 2:1 multiplexer	204.8	2	

**Table 3:** Silicon protection factor (SPF) comparison with state-of-the-art fault-tolerant router architectures.

Fault-Tolerant Router	Area Overhead	Average No. of Defects that Cause Failure	SPF Value
BulletProof [22]	52%	3.15	2.07
VICIS [24]	42%	9.3	6.55
REPAIR [25]	50%	24.5	16.34
SHIELD [28]	34%	15	11.49
HPR [30]	30%	28.5	21.92
Defender [31]	28%	33	25.78
NoCGuard [29]	28%	28.5	22.26
Proposed	26.6%	46.5	36.9

## Abbreviations

The following abbreviations are used in this manuscript:

IoT	Internet of things
NoC	Network-on-chip
ICPS	Industrial cyber–physical systems
MJS	Markov jump systems
HCI	Hot carrier injection
TDDB	Time-dependent dielectric breakdown
NBTI	Negative-bias temperature instability
PEs	Processing elements
RC	Routing computation
VA	Virtual channel allocation
SA	Switch allocation
XBAR	Crossbar
VC	Virtual channel
NMR	N modular redundancy
ECC	Error correcting code
VA1	First stage of VA
VA2	Second stage of VA
SA1	First stage of SA
SA2	Second stage of SA
FIT	Failure in time
MTTF	Mean time to failure
SOFR	Sum of failure rate
FC	Fundamental component
SPF	Silicon protection factor

## References

[1] Zikria Y.B., Afzal M.K., Kim S.W. (2020). Internet of Multimedia Things (IoMT): Opportunities, Challenges and Solutions. Sensors.

[2] Qadri Y.A., Nauman A., Zikria Y.B., Vasilakos A.V., Kim S.W. (2020). The Future of Healthcare Internet of Things: A Survey of Emerging Technologies. IEEE Commun. Surv. Tutorials.

[3] Naeem M.A., Ali R., Alazab M., Yhui M., Zikria Y.B. (2020). Enabling the content dissemination through caching in the state-of-the-art sustainable information and communication technologies. Sustainable Cities Soc..

[4] Yu H., Afzal M.K., Zikria Y.B., Rachedi A., Fitzek F.H. (2020). Tactile Internet: Technologies, test platforms, trials, and applications. Elsevier Future Gener. Comput. Syst..

[5] Yin S., Rodriguez-Andina J.J., Jiang Y. (2019). Real-time monitoring and control of industrial cyberphysical systems: With integrated plant-wide monitoring and control framework. IEEE Ind. Electron. Mag..

[6] Borkar S. Thousand core chips: A technology perspective. Proceedings of the 44th annual design automation conference.

[7] Hoefflinger B. (2011). ITRS: The international technology roadmap for semiconductors. Chips 2020.

[8] Dally W.J., Towles B. Route packets, not wires: On-chip inteconnection networks. Proceedings of the 38th annual Design Automation Conference.

[9] Kumar A. (2017). Intel’s New Mesh Architecture: The ‘Superhighway’of the Data Center. IT Peer Network.

[10] Borkar S. (1999). Design Challenges of Technology Scaling. IEEE Micro.

[11] Kuhn K.J. Reducing variation in advanced logic technologies: Approaches to process and design for manufacturability of nanoscale CMOS. Proceedings of the IEEE International Electron Devices Meeting.

[12] Ziegler J.F. (1998). Terrestrial cosmic ray intensities. IBM J. Res. Dev..

[13] Sai-Halasz G.A., Wordeman M.R., Dennard R.H. (1982). Alpha-particle-induced soft error rate in VLSI circuits. IEEE J. Solid-State Circuits.

[14] Groeseneken G.V. (2001). Hot carrier degradation and ESD in submicrometer CMOS technologies: How do they interact?. IEEE Trans. Device Mater. Reliab..

[15] Oussalah S., Nebel F. On the oxide thickness dependence of the time-dependent-dielectric-breakdown. Proceedings of the IEEE Electron Devices Meeting.

[16] Mahapatra S., Kumar P.B., Dalei T., Sana D., Alam M. Mechanism of negative bias temperature instability in CMOS devices: Degradation, recovery and impact of nitrogen. IEDM Technical Digest. Proceedings of the IEEE International Electron Devices Meeting.

[17] Benini L., De Micheli G. (2002). Networks on chips: A new SoC paradigm. IEEE Comput..

[18] Yang H., Jiang Y., Yin S. (2018). Fault-Tolerant Control of Time-Delay Markov Jump Systems With Ito Stochastic Process and Output Disturbance Based on Sliding Mode Observer. IEEE Trans. Ind. Inf..

[19] Jiang Y., Yin S., Kaynak O. (2018). Data-driven monitoring and safety control of industrial cyber-physical systems: Basics and beyond. IEEE Access.

[20] Hosseinzadeh M., Sinopoli B., Garone E. Feasibility and Detection of Replay Attack in Networked Constrained Cyber-Physical Systems. Proceedings of the 2019 57th Annual Allerton Conference on Communication, Control, and Computing (Allerton).

[21] Tu H., Xia Y., Chi K.T., Chen X. (2020). A Hybrid Cyber Attack Model for Cyber-Physical Power Systems. IEEE Access.

[22] Constantinides K., Plaza S., Blome J., Zhang B., Bertacco V., Mahlke S., Austin T., Orshansky M. BulletProof: A defect-tolerant CMP switch architecture. Proceedings of the IEEE Twelfth International Symposium on High-Performance Computer Architecture.

[23] Kim J., Nicopoulos C., Park D., Narayanan V., Yousif M.S., Das C.R. (2006). A gracefully degrading and energy-efficient modular router architecture for on-chip networks. ACM SIGARCH Comput. Archit. News.

[24] Fick D., DeOrio A., Hu J., Bertacco V., Blaauw D., Sylvester D. Vicis: A reliable network for unreliable silicon. Proceedings of the 46th Annual Design Automation Conference.

[25] Xie L., Mei K., Li Y. Repair: A reliable partial-redundancy-based router in noc. Proceedings of the IEEE eighth international conference on networking, architecture and storage.

[26] Latif K., Rahmani A.M., Nigussie E., Seceleanu T., Radetzki M., Tenhunen H. (2013). Partial virtual channel sharing: A generic methodology to enhance resource management and fault tolerance in networks-on-chip. J. Electron. Test..

[27] Valinataj M., Shahiri M. (2016). A low-cost, fault-tolerant and high-performance router architecture for on-chip networks. Microprocess. Microsyst..

[28] Poluri P., Louri A. (2016). Shield: A reliable network-on-chip router architecture for chip multiprocessors. IEEE Trans. Parallel Distrib. Syst..

[29] Shafique M.A., Baloch N.K., Baig M.I., Hussain F., Zikria Y.B., Kim S.W. (2020). NoCGuard: A Reliable Network-on-Chip Router Architecture. Electronics.

[30] Wang L., Ma S., Li C., Chen W., Wang Z. (2017). A high performance reliable NoC router. Integration.

[31] Baloch N.K., Baig M.I., Daneshtalab M. (2019). Defender: A low overhead and efficient fault-tolerant mechanism for reliable on-chip router. IEEE Access.

[32] Prodromou A., Panteli A., Nicopoulos C., Sazeides Y. Nocalert: An on-line and real-time fault detection mechanism for network-on-chip architectures. Proceedings of the 45th Annual IEEE/ACM International Symposium on Microarchitecture.

[33] Dally W.J., Towles B.P. (2004). Principles and Practices of Interconnection Networks.

[34] Binkert N., Beckmann B., Black G., Reinhardt S., Saidi A., Basu A., Hestness J., Hower D., Krishna T., Sardashti S. (2011). The gem5 simulator. ACM SIGARCH Comput. Archit. News.

[35] Agarwal N., Krishna T., Peh L.S., Jha N.K. GARNET: A detailed on-chip network model inside a full-system simulator. Proceedings of the IEEE international symposium on performance analysis of systems and software.

[36] Woo S.C., Ohara M., Torrie E., Singh J.P., Gupta A. The SPLASH-2 programs: Characterization and methodological considerations. Proceedings of the 22nd Annual International Symposium on Computer Architecture.

[37] Bienia C., Kumar S., Singh J.P., Li K. The PARSEC benchmark suite: Characterization and architectural implications. Proceedings of the 2008 International Conference on Parallel Architectures and Compilation Techniques (PACT).

[38] Martins M., Matos J.M., Ribas R.P., Reis A., Schlinker G., Rech L., Michelsen J. Open cell library in 15nm FreePDK technology. Proceedings of the International Symposium on Physical Design.

[39] Gaver D. (1963). Time to failure and availability of paralleled systems with repair. IEEE Trans. Reliab..

[40] Poluri P., Louri A. An improved router design for reliable on-chip networks. Proceedings of the 28th IEEE International Parallel and Distributed Processing Symposium.

[41] Srinivasan J., Adve S.V., Bose P., Rivers J.A. (2004). The case for lifetime reliability-aware microprocessors. ACM SIGARCH Comput. Archit. News.

[42] Trivedi K.S. (1982). Probability and statistics with reliability, queuing, and computer science applications.

